# Cardiac Complications From Herbal Remedies

**DOI:** 10.7759/cureus.76213

**Published:** 2024-12-22

**Authors:** Hakob Harutyunyan, Vahagn Tamazyan, Aleksan Khachatryan, Ashot Batikyan, Prabal KC, Surik Sedrakyan, Xixi Yin

**Affiliations:** 1 Department of Internal Medicine, Maimonides Medical Center, New York, USA; 2 Department of Internal Medicine, University of Maryland Medical Center, Midtown Campus, Baltimore, USA; 3 Department of Internal Medicine, North Central Bronx Hospital, New York, USA; 4 Department of Internal Medicine, St. Elizabeth’s Medical Center, Boston, USA; 5 Department of Cardiology, Maimonides Medical Center, New York, USA

**Keywords:** arrhythmia induced cardiomyopathy, caffeine, herbal medicine, tachycardia-induced cardiomyopathy, traditional chinese medicine

## Abstract

The safety of traditional Chinese medicine (TCM) herbal remedies, particularly when used with modern medications or in non-traditional dosages, requires careful consideration. We present a case of a 62-year-old male with pre-existing cardiovascular risk factors who developed tachycardia-induced cardiomyopathy (TIC) potentially linked to prolonged use of the TCM supplement "Tan Ke Jing." The supplement contains licorice root, caffeine, and apricot kernel, which have known cardiovascular effects. Discontinuation of the supplement and guideline-directed medical therapy significantly improved left ventricular ejection fraction over four months. This case highlights the potential for TCM herbal supplements to induce serious cardiac conditions, emphasizing the importance of thorough patient history taking, including alternative medicine use, and the need for further research into interactions and side effects. Clinicians should be aware of the cardiac risks associated with herbal supplement use and foster open communication with patients about alternative medicine use.

## Introduction

Traditional Chinese Medicine (TCM) is a respected therapeutic tradition with a rich history of offering medications derived from a synergy of herbs, roots, and natural elements. While TCM has relieved many, its safety profile, particularly in conjunction with modern medications or when shifting from traditional dosages, demands a nuanced understanding. Current evidence highlights that Chinese herbal medicines, once deemed benign, are not without risk, with documented fatalities emphasizing the need for an attentive approach [1]. Within this context, we present a case of persistent tachycardia leading to tachycardia-mediated cardiomyopathy (TMC) in a patient undertaking prolonged consumption of the TCM herbal supplement "Tan Ke Jing." This formulation, prepared to maintain a healthy throat and bronchial system, contains an herbal ensemble including Gan Cao (Glycyrrhiza glabra or licorice root), Ka Fei Yin (caffeine), Yuan Zhi, Bing Pian, Ku Xing Ren, Wu Bei Zi, and Jie Geng [1]. This report shows potential links between the herb's components and the emergence of critical cardiac symptoms in the patient. Doing so emphasizes the importance of understanding the potential side effects and interactions in TCM formulations.

## Case presentation

**Figure 1:**
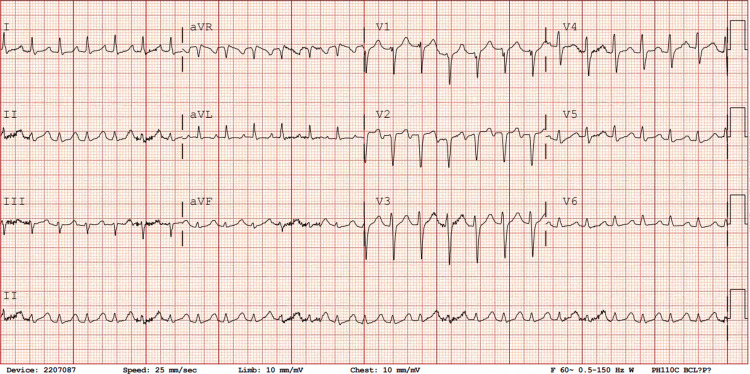
Initial electrocardiogram Shows tachycardia around 200 bpm with narrow QRS complexes (< 120ms) consistent with supraventricular tachycardia (SVT).

A 62-year-old Chinese male presented with an acute onset of shortness of breath. His past medical history includes hypertension, diabetes mellitus, hyperlipidemia, active smoking, and a cerebrovascular accident three months prior, which resulted in speech impairment and left-sided weakness. The patient's acute respiratory distress began while he was showering. During the EMS assessment, he exhibited increased work of breathing, diaphoresis, and low oxygen saturation (SPO2). Nitroglycerin was administered en route to the hospital, and Continuous Positive Airway Pressure therapy was initiated. SPO2 was in the 80s upon arrival at the Emergency Department (ED). During the initial evaluation in the ED, the patient was in significant respiratory distress. Physical examination revealed scattered crackles in both lungs, tachycardia with normal heart sounds, and trace edema in the lower extremities. Vital signs showed SPO2 of 82% on room air, blood pressure (BP) of 190/110 mmHg, and an electrocardiogram (ECG) revealed supraventricular tachycardia (SVT) with a heart rate of 150 beats per minute (Figure 1).

**Figure 2:**
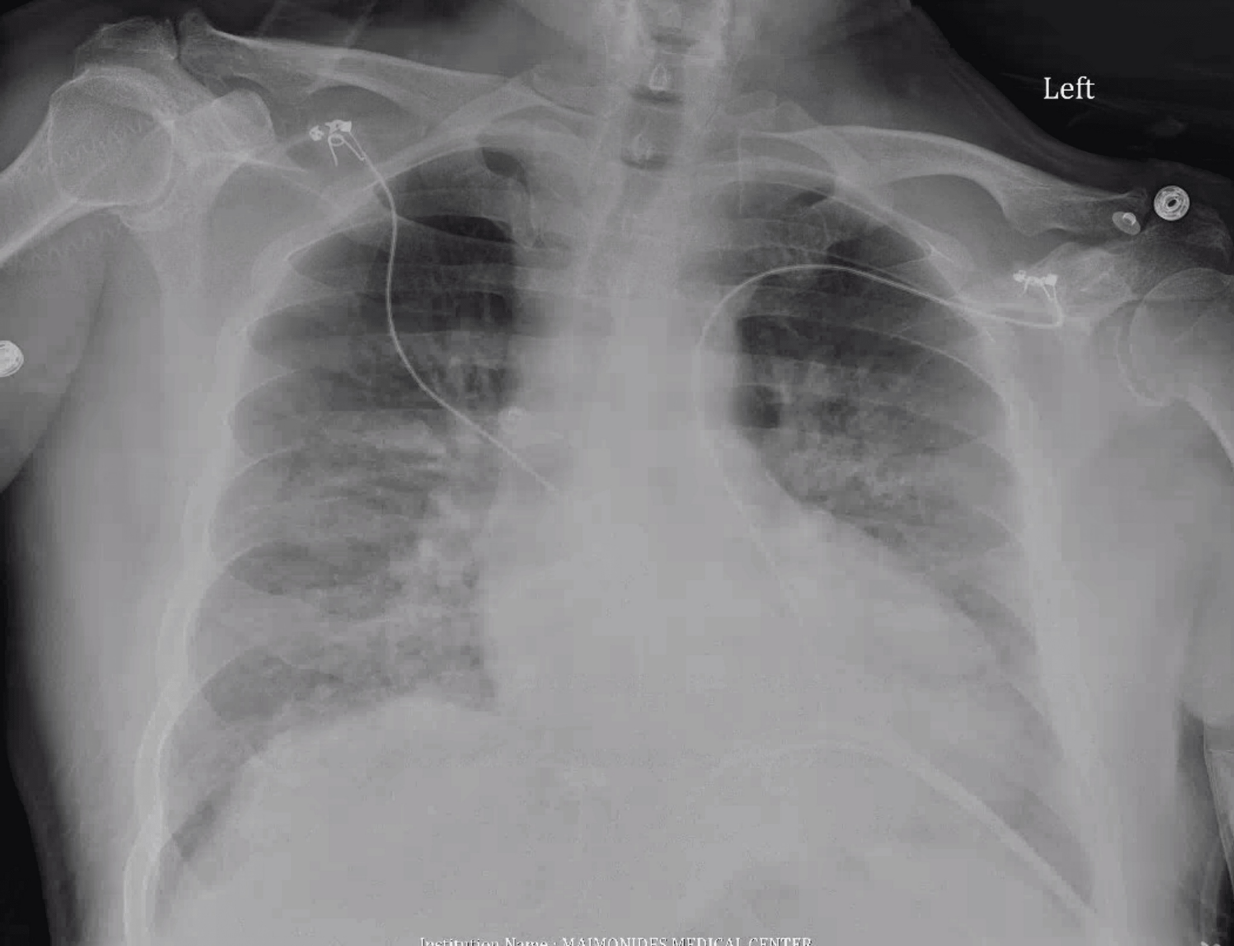
Chest X-ray Anterioposterior chest X-ray suggestive of pulmonary congestion/edema.

Pertinent laboratory results included an initially low troponin level of 0.03 ng/ml (later increased to 0.09 ng/ml), creatinine of 1.5 mg/dl, an elevated B-type natriuretic peptide (BNP) of 1081 pg/mL, thyroid stimulating hormone (TSH) of 1.39 mciu/ml, and a D-dimer level of 347 ng/mL. Imaging studies demonstrated indications of fluid overload on Chest X-ray (CXR) (Figure 2), diffuse B-lines with a globally decreased ejection fraction (EF) on point-of-care ultrasonography (POCUS), absent pericardial effusion, and bilateral pleural effusions. An official echocardiogram revealed global cardiomyopathy with a significantly reduced left ventricular ejection fraction (LVEF) of 26%.

**Figure 3:**
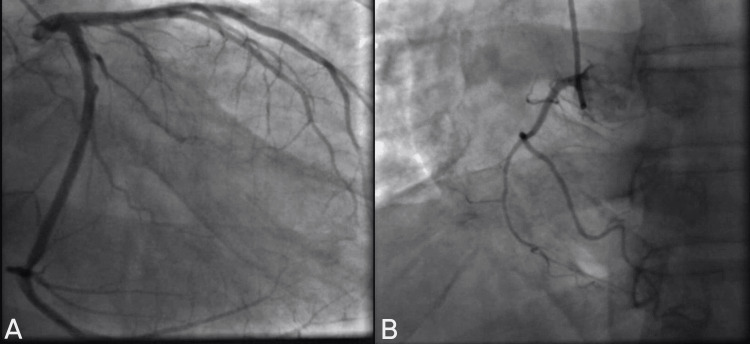
Coronary angiography of the left (A) and right coronary artery (B) branches There are no hemodynamically significant coronary artery lesions.

Despite initial concerns for acute coronary syndrome (ACS) given the mild troponin elevation, left heart catheterization (LHC) ruled out significant coronary artery disease (Figure 3).

Further discussion revealed the patient’s use of Ke Tan Jing powder daily to reduce sputum. He admitted to frequent palpitations but was unaware of the potential cardiovascular implications of this herbal supplement. Hospital management included diuresis, leading to notable improvement in his respiratory status. He was started on guideline-directed medical therapy (GDMT), including carvedilol and sacubitril/valsartan (Entresto). He was advised to discontinue Ke Tan Jing due to its suspected contribution to his cardiomyopathy. A repeat echocardiogram after four months showed significant improvement in LVEF from 26% to 51-55%.

## Discussion

Individuals with heart failure (HF) and impaired left ventricular (LV) function frequently coexist with arrhythmias. Premature ventricular contractions, atrial fibrillation, and tachycardias are known to cause arrhythmia-induced cardiomyopathy (AiCM), a potentially reversible dilated cardiomyopathy. The main challenge is determining how much- if at all, partially, or entirely- arrhythmias are to blame for the reported LV dysfunction. Patients with a mean heart rate greater than 100 beats per minute, atrial fibrillation, and/or a premature ventricular contraction burden greater than 10% should be evaluated for AiCM. When the cardiomyopathy reverses, and the arrhythmia is eliminated, this confirms the existence of AiCM. The diagnosed arrhythmia, the patient's comorbidities, and personal preferences all influence the therapeutic decision. Patients require continuous monitoring once LV function returns, mainly if an aberrant myocardial substrate is present [2]. In the case of the 62-year-old male patient who presented with sinus tachycardia, respiratory distress, and evidence of cardiomyopathy with reduced LV ejection fraction, an interesting finding was his daily consumption of Tan Ke Jing, a traditional Chinese medicine. Tan Ke Jing is an herbal supplement that helps maintain a healthy throat and bronchial system. During the consumption of Tan Ke Jing, it is advised to refrain from smoking and alcohol consumption and intake of spicy, raw, cold, and greasy foods. It's important to note that this product is not intended for long-term use. While the direct etiology of his cardiomyopathy remains complex, a deeper examination of the ingredients of this herbal preparation might shed light on potential exacerbating factors.

Ka Fei Yin (caffeine)

As a central nervous system stimulant, caffeine's cardiac effects are extensively documented. In elevated amounts, it can lead to palpitations and tachycardia, and potentially even predispose one to various cardiac arrhythmias. If consumed excessively over an extended period, the consequent tachycardia could contribute to the development or exacerbation of cardiomyopathy [3,4].

Gan Cao (Glycyrrhiza glabra or licorice root)

Gan Cao contains the compound glycyrrhizin, which has the potential to mimic the effects of aldosterone. This pseudo-hypoaldosteronism can lead to sodium retention and potassium loss, resulting in electrolyte imbalance. This imbalance may induce premature ventricular contractions (PVC), atrial premature contractions (APCs), and supraventricular and ventricular tachycardias. Additionally, it has the potential to contribute to hypertension. If left unaddressed, the chronic effects of hypertension may predispose the myocardium to hypertrophy and eventual cardiomyopathy [5-7].

Ku Xing Ren (Prunus armeniaca or apricot kernel)

A less direct but equally crucial connection to consider is the potential release of cyanide from amygdalin found in apricot kernels. While not directly leading to cardiomyopathy, cyanide poisoning can have multiple systemic effects, including on the cardiovascular system [8,9].

While the above components are the most directly connected to potential cardiac effects, it's critical to recognize that herbal preparations' safety and efficacy often lie in their synergistic actions. The interactions between these ingredients or other medications or conditions the patient may have could result in unexpected effects. The holistic view of such traditional therapies often contributes to their therapeutic action, but it can also complicate understanding potential side effects. TMC may result from supraventricular or ventricular tachycardias or frequent premature ventricular contractions (PVCs). The time to develop cardiomyopathy is not well-established, but it may develop within days to months of tachycardia onset. Cardiomyopathy may develop when the PVC burden is greater than 10,000 per day or more than 10% of all beats. The hallmark of TMC is that it is reversible with correction of the tachycardia. Successful treatment with either rate or rhythm control improves or corrects cardiomyopathy [2]. In the context of our patient, it's vital to probe further the dosage and duration of his Tan Ke Jing consumption. The long-standing daily intake contributed cumulatively to his presentation, particularly in an individual with pre-existing cardiovascular conditions. Given the global popularity of traditional and herbal medicines, understanding and researching their potential interactions with conventional therapies and their direct effects becomes vital. As clinicians, encouraging a non-judgmental dialogue with patients about their use of such remedies is crucial. This case stresses this significance, revealing the potentially significant cardiac effects that might go unrecognized in the absence of such a conversation.

## Conclusions

This case demonstrates the importance of awareness in integrative medicine. The patient's experience, marked by persistent tachycardia and cardiomyopathy, illustrates the complex relationship between traditional herbal formulations and cardiovascular health. The ingredients in Tan Ke Jing - caffeine, licorice root, and apricot kernel - likely contributed to the patient's cardiac presentation. This case shows that understanding herbal preparations' synergistic actions and cumulative effects is necessary beyond examining individual isolated components. The patient's cardiomyopathy significantly improved after starting GDMT and discontinuing Tan Ke Jing, demonstrating the dynamic nature of TMC. Clinicians should recognize that TMC can be reversible and promptly address the underlying tachyarrhythmia. As traditional and herbal medicines gain popularity worldwide, understanding their interactions with conventional therapies becomes increasingly essential. Clinicians should encourage open and non-judgmental discussions with patients about using these therapies. This case report illustrates the potential for significant cardiac effects and advocates for ongoing research, awareness, and collaborative patient-clinician dialogues in the complex landscape of integrative medicine.
